# Salmonella Typhi Osteomyelitis of the Ulnar Diaphysis in an Immunocompetent Adult: A Case Report

**DOI:** 10.7759/cureus.111132

**Published:** 2026-06-19

**Authors:** Shashank Singh, R. Maheshwari

**Affiliations:** 1 Orthopedics, Swami Rama Himalayan University, Dehradun, IND

**Keywords:** case report, chronic osteomyelitis, enteric fever, fluoroquinolone resistance, osteomyelitis, salmonella typhi, ulna

## Abstract

*Salmonella Typhi* is an uncommon cause of chronic osteomyelitis and may present diagnostic challenges, particularly in patients without identifiable predisposing factors. We report the case of a 22-year-old immunocompetent man who presented with progressive pain and swelling over the middle third of the left forearm for four months. Laboratory investigations revealed normal inflammatory markers, including an erythrocyte sedimentation rate of 2 mm/hr and a C-reactive protein level of <0.50 mg/dL. Plain radiographs demonstrated cortical thickening, periosteal reaction, and sequestrum formation involving the ulnar shaft, while MRI showed chronic intramedullary osteomyelitis with cortical cloaca formation and an intracortical abscess. The patient underwent surgical sequestrectomy and debridement. Intraoperative bone cultures grew *S*. Typhi resistant to ciprofloxacin, levofloxacin, and azithromycin but sensitive to ceftriaxone and cotrimoxazole. Histopathological examination confirmed chronic osteomyelitis. Treatment with intravenous ceftriaxone followed by oral cotrimoxazole resulted in complete clinical and radiological recovery without recurrence. This case emphasizes the importance of obtaining tissue cultures in chronic osteomyelitis to identify unusual pathogens and guide appropriate antimicrobial therapy. *Salmonella* Typhi should be considered among the potential etiological agents of chronic osteomyelitis, even in the absence of recognized risk factors.

## Introduction

Osteomyelitis is most commonly caused by *Staphylococcus aureus*, whereas Gram-negative organisms account for a much smaller proportion of cases [1]. Salmonella species are rare causes of bone infection and are classically associated with sickle cell disease, hemoglobinopathies, immunosuppression, malignancy, and chronic systemic illnesses [2]. Although *Salmonella* Typhi is endemic in many developing countries and remains a significant cause of enteric fever, skeletal involvement occurs infrequently [3].

The pathogenesis of *Salmonella* osteomyelitis is believed to involve hematogenous dissemination during bacteremia with subsequent localization within bone [4]. The vertebrae, femur, tibia, and humerus represent the most commonly reported sites [5,6]. Involvement of the ulnar diaphysis is exceedingly uncommon, especially in immunocompetent adults.

Because clinical presentation is often indolent and laboratory markers may be normal, diagnosis can be delayed or mistaken for neoplastic or other infectious conditions. We report a rare case of culture-proven *S. *Typhi osteomyelitis involving the mid-diaphysis of the ulna in an immunocompetent adult, emphasizing the diagnostic challenges, importance of microbiological confirmation, and implications of emerging antimicrobial resistance.

## Case presentation

A 22-year-old previously healthy male presented with a four-month history of insidious-onset pain and swelling involving the middle third of the left forearm. There was no preceding history of local trauma, surgical intervention, intravenous drug use, or known immunocompromising conditions.

The clinical history points to a distinct prior exposure: the patient recalled a self-limiting febrile illness approximately six weeks prior to symptom onset that had been managed conservatively at a peripheral center without microbiological investigation. In retrospective clinical correlation, this febrile episode represented the primary systemic bacteremic phase of enteric fever, during which transient hematogenous seeding occurred within the ulnar diaphysis. While his immune response successfully cleared the systemic gastrointestinal and vascular infection, the pathogen found an immunologically privileged niche within the bone, surviving intracellularly to form a localized, chronic nidus.

On physical examination, the patient was afebrile and hemodynamically stable, showing no systemic signs of toxicity. Local examination revealed a localized swelling over the medial aspect of the middle third of the left forearm, corresponding to the ulnar shaft. The swelling was tender, non-mobile, and hard in consistency, with no overlying erythema, warmth, active sinus formation, or skin changes. Movements of the elbow, wrist, and forearm were painless and unrestricted, and the distal neurovascular examination was completely normal (Figure 1).

**Figure 1:**
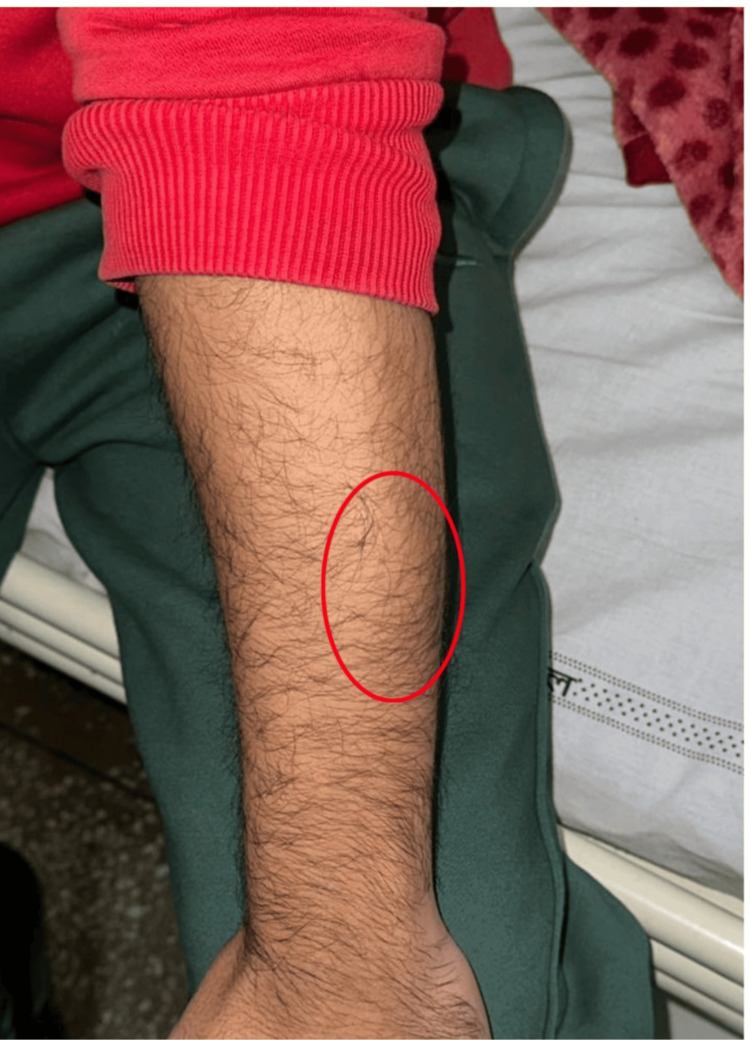
Preoperative clinical photograph demonstrating localized swelling over the medial surface of the middle-third of the left forearm

Diagnostic assessment

Systemic Screening and Carrier Status Evaluation

To evaluate for systemic dissemination, active bacteremia, or a potential healthcare-associated infection (HAI), a comprehensive systemic workup was performed at admission. Paired sets of peripheral blood cultures and a clean-catch midstream urine culture were obtained; all systemic cultures yielded no growth after prolonged incubation, ruling out concurrent bacteremia or active sepsis. Given the four-month duration of localized symptoms prior to admission, the presentation was classified as a community-acquired, post-infectious focal sequela of enteric fever rather than a nosocomial or hospital-acquired process.

Furthermore, post-treatment evaluation included sequential stool cultivation to assess for chronic carrier status. Chronic gastrointestinal carriage of *S.* Typhi is defined as the persistence of the organism in stool or urine for more than 12 months. The patient's post-treatment stool cultures were completely negative, ruling out persistent colonization of the gallbladder or intestinal mucosa, confirming that the infection was entirely an isolated, sequestered chronic osteomyelitis rather than a chronic shedding state. The patient's laboratory investigations at presentation are summarized in Table 1. Notably, inflammatory markers were within normal limits despite the presence of chronic osteomyelitis.

**Table 1:** The patient's laboratory investigations at presentation

Parameter	Result	Unit	Reference Range
Hemoglobin	14.7	g/dL	13.0-17.0
Total leukocyte count	5,800	cells/mm³	4,000-11,000
Erythrocyte sedimentation rate (ESR)	2	mm/hr	0-15
C-reactive protein (CRP)	<0.50	mg/dL	<0.50
Serum creatinine	1.06	mg/dL	0.7-1.3

Despite the chronicity and radiological severity of the bone lesion, acute-phase reactants and inflammatory markers remained completely within normal physiological limits. This highlights that acute inflammatory markers are highly unreliable and do not necessarily elevate or change in chronic, well-sequestered conditions.

Advanced Imaging and Radiologist Commentary

Plain radiographs demonstrated soft-tissue swelling, marked cortical thickening, periosteal reaction, and clear sequestrum formation involving the mid-diaphysis of the left ulna. Advanced cortical thickening and reactive sclerosis reflect a prolonged, low-grade periosteal response to intraosseous tension. The presence of a distinct, dense, avascular bone fragment, i.e., the sequestrum, confirmed focal cortical necrosis where the local blood supply was entirely compromised by the infection (Figure 2).

**Figure 2:**
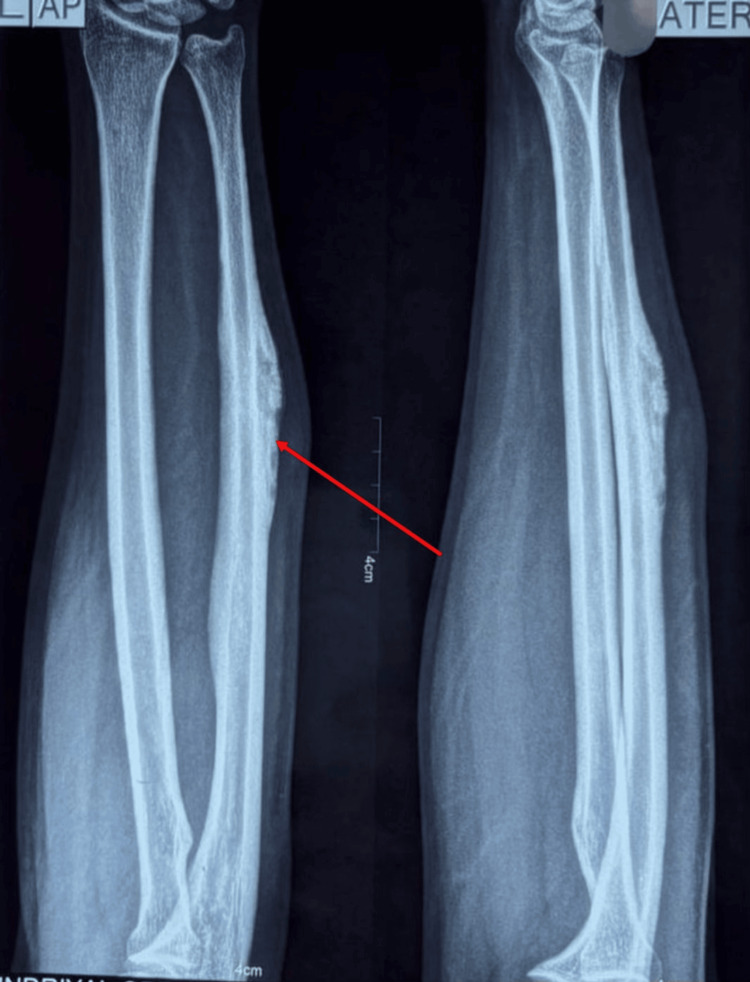
Preoperative anteroposterior and lateral radiographs of the left forearm demonstrating soft tissue swelling, marked cortical thickening, and periosteal reaction of the ulnar diaphysis (red arrow)

The MRI of the forearm demonstrated diffuse intramedullary T2/short tau inversion recovery (STIR) hyperintensity extending approximately 7.6 cm within the ulnar shaft with post-contrast enhancement, pinpointing active bone marrow edema consistent with active osteomyelitis. A well-defined intracortical abscess measuring 5 X 6 X 19 mm was identified. The identification of this abscess breaching the cortex via defined tracking channels, multiple cortical cloacae communicating with surrounding soft tissues, provided the classic radiological fingerprint of a chronic process. This structural breakdown allowed intraosseous pus to decompress into the surrounding soft tissues, forming an adjacent subcutaneous fluid collection (Figures 3-5).

**Figure 3:**
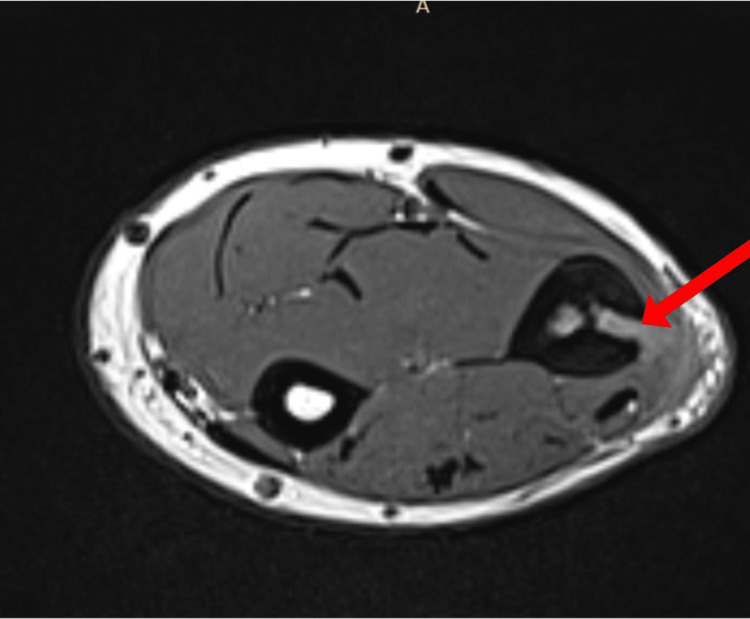
Axial T2/STIR MRI sequences demonstrating a cloaca extending through the lateral cortex (red arrow) STIR: Short tau inversion recovery

**Figure 4:**
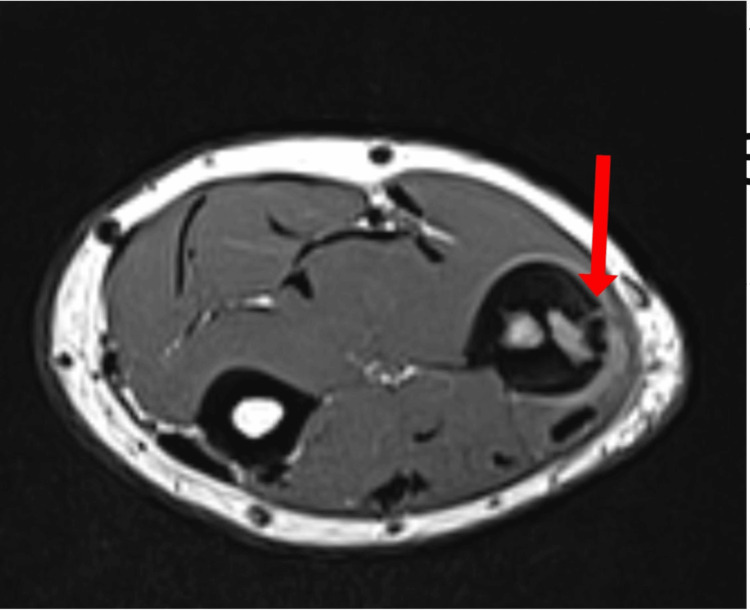
Axial T2/STIR MRI sequences demonstrating marked cortical thickening and reactive sclerosis (red arrow) STIR: Short tau inversion recovery

**Figure 5:**
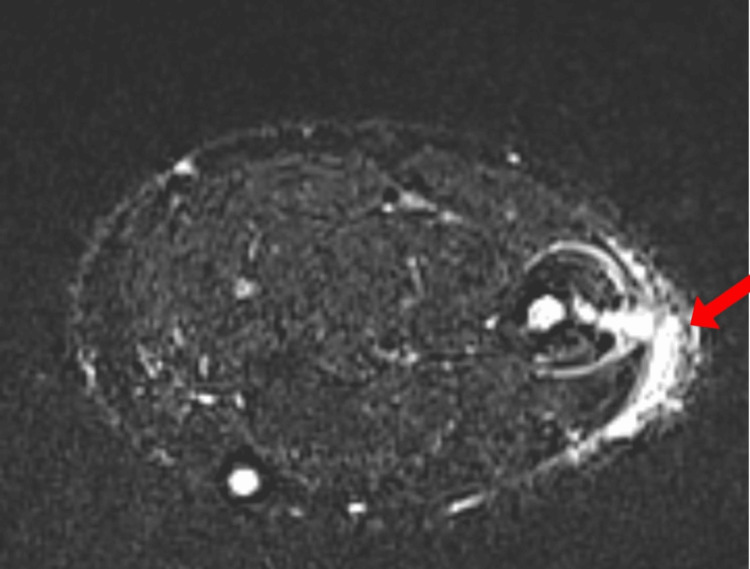
Axial T2/STIR MRI sequences demonstrating an adjoining subcutaneous fluid collection/abscess (red arrow) STIR: Short tau inversion recovery

Therapeutic intervention and clinical microbiology protocol

Surgical Sampling and Anaerobic Precautions

Considering the chronic nature of the lesion and radiological evidence of sequestrum formation, the patient was scheduled for surgical debridement, cortical windowing, curettage, and sequestrectomy. To ensure optimal microbiological yield and prevent diagnostic interference, all empirical antibiotic therapy and standard preoperative surgical antibiotic prophylaxis were strictly withheld until deep tissue samples were successfully harvested.

Following surgical exposure and identification of the cortical opening (cloaca) (Figure 6), superficial swabs were intentionally avoided to eliminate the risk of contamination by skin commensals. Instead, under strict aseptic conditions, multiple deep tissue biopsies and bone fragments were harvested directly from the intramedullary canal and the margins of the viable/necrotic bone interface.

**Figure 6:**
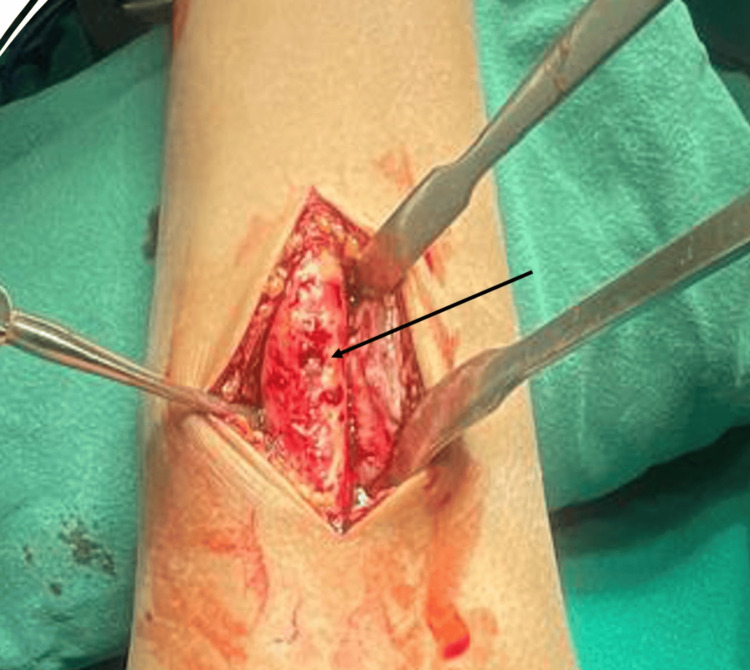
Intraoperative photograph demonstrating the cortical opening (cloaca) following surgical exposure

To preserve fastidious and obligate anaerobic organisms, which are critically important in the differential diagnosis of chronic orthopedic infections, the harvested deep tissue fragments were immediately transferred into oxygen-free anaerobic transport media containers at the surgical field. The specimens were delivered to the clinical microbiology laboratory within 45 minutes of collection for immediate processing, aerobic/anaerobic cultivation, and histopathological analysis. Immediate postoperative radiographs demonstrated satisfactory cortical windowing and removal of necrotic bone (Figure 7).

**Figure 7:**
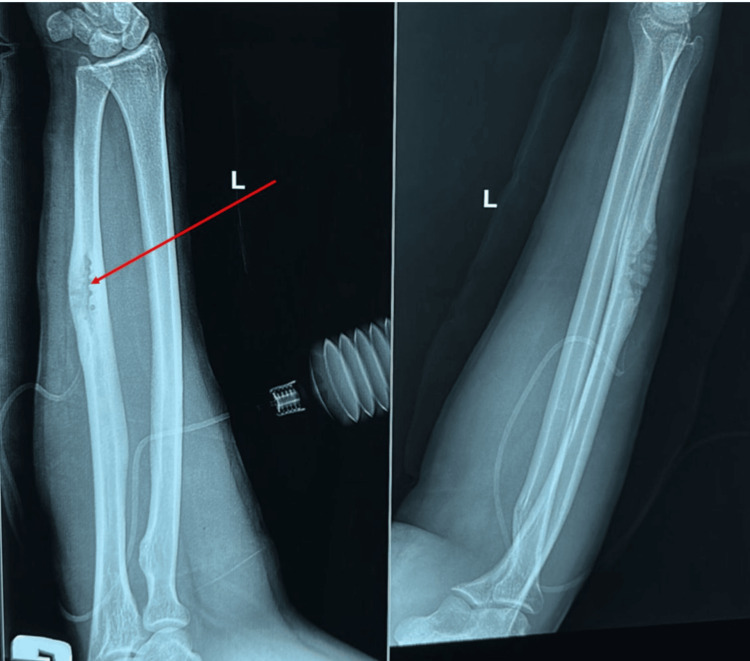
Immediate postoperative anteroposterior and lateral radiographs following extensive sequestrectomy and cortical windowing

Microbiological Identification and Cascade Antimicrobial Susceptibility Testing (AST)

Under a laminar flow hood, deep bone fragments were homogenized in sterile saline and inoculated onto aerobic and anaerobic cultivation media. Smooth, non-lactose-fermenting colonies isolated after 24 hours of incubation at 37°C were definitively identified as *S.* Typhi via automated profiling and confirmed through specific serological agglutination.

An AST was performed using automated broth microdilution to determine exact minimum inhibitory concentrations (MICs), interpreted strictly according to contemporary Clinical and Laboratory Standards Institute (CLSI) guidelines. In accordance with institutional antibiotic stewardship protocols, results were released using a tier-wise cascade reporting protocol to guide narrow-spectrum targeting and preserve reserve agents. The complete antimicrobial susceptibility profile is presented in Table 2.

**Table 2:** Intraoperative deep bone culture susceptibility and cascade reporting MIC: Minimum inhibitory concentration, q12h: Every 12 hours

Testing tier	Antimicrobial agent	MIC	Clinical interpretation	Actionable stewardship and treatment rationale
Tier 1 (first-line/standard)	Ampicillin, cotrimoxazole, ciprofloxacin, levofloxacin	—, <=20 µg/mL, >=4 µg/mL, >=8 µg/mL	Sensitive, sensitive, resistant, resistant	*S.* Typhi isolates in South Asia show extensive fluoroquinolone resistance. High-level resistance triggered automatic escalation to tier 2 protocols.
Tier 2 (escalated agents)	Ceftriaxone, ceftazidime, azithromycin	—, 0.25 µg/mL, —	Sensitive, sensitive, resistant	Selected line of therapy: Intravenous ceftriaxone (2 g q12h for two weeks) was initiated due to excellent cortical bone penetration, followed by oral cotrimoxazole for four weeks
Tier 3 (restricted/reserve)	Imipenem, meropenem	<=0.25 µg/mL, <=0.25 µg/mL	Sensitive, sensitive	Protected stewardship agents: Kept hidden from active clinical view and spared completely to prevent emerging carbapenem resistance

Bone Pathology Analysis

Histopathological examination of the decalcified intraoperative bone specimens provided tissue-level confirmation and ruled out malignant or granulomatous mimics. Microscopic examination revealed a complete disruption of normal architecture, characterized by a mixture of viable bony lamellae (with intact osteocytes within lacunae) and irregular, acellular necrotic bony lamellae (sequestrum) lacking osteocytes. The surrounding fibrocollagenous stroma exhibited marked edema, active hyperemia, and a prominent chronic inflammatory infiltrate rich in lymphocytes and plasma cells, accompanied by focal neutrophilic infiltration. The total absence of epithelioid granulomas, Langhans giant cells, or caseous necrosis ruled out tubercular osteomyelitis, while the absence of cellular atypia or abnormal mitotic figures ruled out osteosarcoma or Ewing's sarcoma (Figure 8).

**Figure 8:**
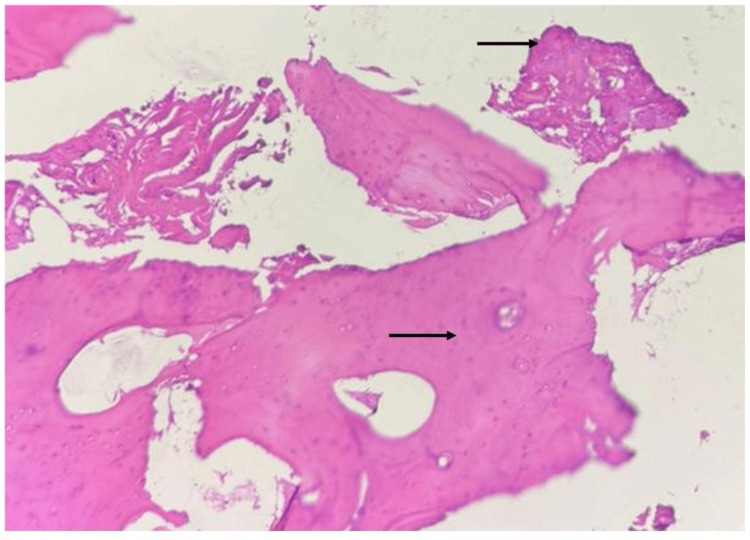
Postoperative histopathology slide (H&E stain, 100x magnification) revealing a mixture of viable and necrotic bony lamellae surrounded by dense chronic inflammatory infiltrate (black arrows)

Postoperative course and targeted outcome linkage

The successful eradication of this infection without recurrence was the direct result of pairing targeted, culture-guided antimicrobials with aggressive mechanical debridement. *Salmonella* Typhi creates dense biofilms within avascular necrotic bone. Because systemic antibiotics cannot penetrate unvascularized tissue, surgical debridement and sequestrectomy were mandatory to mechanically eliminate the bacterial nidus.

Following debridement, initiating intravenous ceftriaxone (2g twice daily) for two weeks took advantage of the drug’s high cortical bone penetration, successfully sterilizing the surrounding hyperemic bone margins. Transitioning to oral cotrimoxazole for an additional four weeks provided sustained consolidative therapy to eliminate any remaining intracellular pathogens surviving within local macrophages. At the final follow-up, clinical examination demonstrated complete resolution of swelling and a well-healed surgical incision (Figure 9), while follow-up radiographs showed progressive bone consolidation without evidence of recurrent infection (Figure 10). Stool culture and sickling tests were negative. A summary of the patient's clinical course is provided in Table 3. This case report has been prepared in accordance with the CARE reporting guidelines.

**Figure 9:**
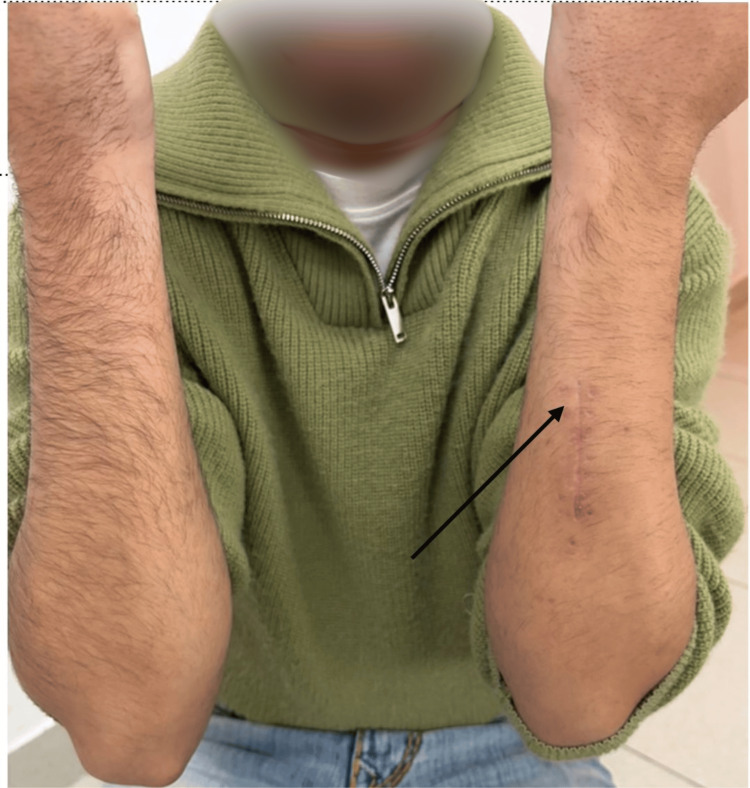
Six-week postoperative follow-up demonstrating a well-healed surgical incision with full clinical resolution

**Figure 10:**
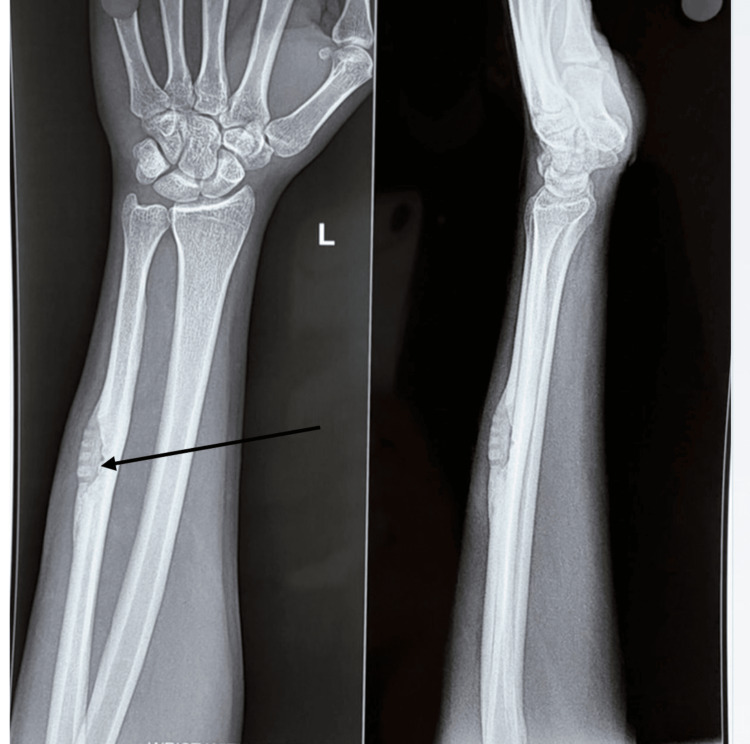
Corresponding radiographs showing progressive bone consolidation

**Table 3:** Timeline of the patient's journey to recovery

Time	Event
Six weeks before symptoms	Febrile illness
Four months	Forearm pain and swelling
Presentation	Imaging and laboratory evaluation
Surgery	Debridement and sequestrectomy
Culture result	*S.* Typhi isolated
Two weeks	IV ceftriaxone
Six weeks	Clinical and radiological recovery

## Discussion

*Salmonella* osteomyelitis remains an uncommon clinical entity despite the widespread prevalence of enteric fever. Historically, the condition has been strongly associated with sickle cell disease because repeated vaso-occlusive crises create infarcted bone susceptible to bacterial seeding [2]. However, recent literature suggests that a substantial proportion of cases now occur in patients without traditional risk factors, particularly in regions where typhoid fever remains endemic [5,6].

The pathogenesis involves hematogenous dissemination during transient bacteremia [4]. Following entry into the bloodstream, *Salmonella* organisms can survive intracellularly within macrophages and subsequently localize within bone tissue [2]. In chronic cases, sequestrum formation, cortical destruction, and cloaca formation may occur, resulting in persistent infection despite minimal systemic manifestations [4].

One of the most notable features of the present case was the absence of elevated inflammatory markers. The patient demonstrated normal leukocyte count, ESR, and CRP despite radiological evidence of extensive chronic osteomyelitis. Similar observations have been reported in previous cases of indolent* Salmonella* osteomyelitis, illustrating the diagnostic challenge posed by these infections and highlighting the limitations of relying solely on laboratory markers [7,8]. 

Radiologically, chronic osteomyelitis caused by *Salmonella* is indistinguishable from infections caused by more common organisms. The MRI remains the most sensitive imaging modality for identifying intramedullary infection, cortical breaches, abscess formation, and soft-tissue extension. Nevertheless, imaging alone cannot establish a microbiological diagnosis.

A definitive diagnosis requires tissue sampling. Blood cultures are frequently negative in chronic presentations because bacteremia has often resolved by the time musculoskeletal symptoms develop. Consequently, deep tissue or bone cultures remain the diagnostic gold standard for pathogen identification and antimicrobial susceptibility testing [1,2,9]. In the present case, culture-directed therapy was crucial because empirical treatment may not have adequately addressed the observed antimicrobial resistance profile.

The emergence of fluoroquinolone-resistant *S.* Typhi strains has become a significant public health concern throughout South Asia. Resistance to ciprofloxacin and levofloxacin, as observed in this patient, increasingly limits therapeutic options and reinforces the need for sensitivity-guided treatment [10]. Third-generation cephalosporins and cotrimoxazole remain effective alternatives in many cases.

Recent epidemiological studies have demonstrated an increasing prevalence of multidrug-resistant and fluoroquinolone-resistant *S.* Typhi strains across South Asia. This trend has important implications for musculoskeletal infections, where empirical fluoroquinolone therapy may lead to treatment failure. Culture-guided antimicrobial selection, therefore, remains essential, particularly in endemic regions where resistance patterns continue to evolve [11]. 

The anatomical location further contributes to the uniqueness of this report. Most published cases involve the vertebrae, femur, tibia, or humerus [2,5,6] (Table 4). Isolated involvement of the ulnar diaphysis in an immunocompetent adult has rarely been described, with the most comparable report being the Brodie’s abscess case described by Ip et al. [12]. Awareness of such atypical presentations may facilitate earlier diagnosis and treatment.

**Table 4:** Reported upper-limb Salmonella osteomyelitis cases compared with the present case

Author and reference no.	Year	Site involved	Host status	Salmonella species	Management	Outcome
Ip et al. [12]	2008	Ulna (Brodie's abscess)	Immunocompetent adult female	S. Typhi	CT-guided biopsy, surgical debridement, and antibiotics	Recovered
Itoh et al. [13]	2021	Humerus	Immunocompetent adult male	Salmonella enterica	Biopsy, curettage, and antibiotics	Recovered
Willen et al. [14]	2021	Chronic osteomyelitis of a long bone	Immunocompetent adult male	Salmonella spp.	Surgical debridement and antibiotics	Recovered
Venkataram et al. [15]	2020	Long bone with a pathological fracture	Immunocompetent adult	S. Typhi	Debridement, stabilization, and antibiotics	Recovered
Present case	2026	Mid-diaphyseal ulna with cloaca and sequestrum	Immunocompetent adult male	S. Typhi	Sequestrectomy, cortical windowing, and culture-directed ceftriaxone followed by cotrimoxazole	Recovered

A review of the available literature demonstrates that *Salmonella *osteomyelitis in immunocompetent adults remains exceedingly uncommon. Reported cases most frequently involve the vertebrae and long bones of the lower extremity, whereas upper-limb involvement is rare [2,5,6]. Among published reports, isolated ulnar involvement has been described only sporadically, most notably as a Brodie’s abscess reported by Ip et al. [12]. Other upper extremity long bone involvements mimicking malignancies or presenting with pathological fractures have been documented (Table 4) by Itoh et al. [13], Willen et al. [14], and Venkataram et al. [15]. In comparison, the present case demonstrated chronic diaphyseal osteomyelitis with sequestrum formation, multiple cloacae, and microbiologically confirmed fluoroquinolone-resistant *S.* Typhi, highlighting both the unusual anatomical location and the contemporary challenge of antimicrobial resistance [12-17]. From an expert microbiological perspective, three critical factors must be highlighted to guide surgical and medical management.

One, the intracellular privilege and diagnostic failure of biomarkers;* S. *Typhi is a facultative intracellular pathogen. By surviving inside host macrophages and sequestering within avascular necrotic bone, the organism effectively isolates itself from systemic immune surveillance. This explains why standard acute inflammatory markers (ESR, CRP) are highly unreliable in these chronic conditions and fail to elevate. A normal inflammatory panel must never be used to rule out osteomyelitis when atypical Gram-negative pathogens are in the differential diagnosis.

Two, critical specimen logistics over empirical prophylaxis. In chronic orthopedic infections, the temptation to initiate broad-spectrum empirical therapy or administer routine preoperative surgical prophylaxis prior to incision can completely sterilize fastidious cultures, leading to diagnostic failure. Securing multiple deep-seated intraoperative tissue and bone biopsies under strict aseptic conditions, while intentionally bypassing superficial tract swabs, is the absolute gold standard for pathogen identification. Furthermore, immediate placement into anaerobic transport media is vital to rule out polymicrobial or fastidious anaerobic co-infections common in chronic bone lesions.

Three, the alarm of fluoroquinolone resistance and stewardship. For decades, fluoroquinolones were the frontline oral agents for bone infections due to their excellent cortical penetration. The high-level resistance demonstrated by this isolate mirrors a dangerous, expanding trend of multidrug-resistant and fluoroquinolone-resistant *S.* Typhi clones across South Asia. This underscores the absolute necessity of institutional cascade reporting protocols in clinical microbiology labs. By suppressing reserve agents (tier 3 carbapenems) from the initial clinician report, microbiological stewardship successfully drove the utilization of targeted tier 2 agents, achieving complete clinical eradication without compounding global carbapenem resistance.

Clinical relevance, novelty, and literature gap

Orthopedic surgeons should maintain a high index of suspicion for atypical pathogens in chronic osteomyelitis. Tissue biopsy and microbiological analysis are indispensable when clinical and laboratory findings are inconclusive. This case documents* S.* Typhi osteomyelitis in an unusual anatomical location, namely the ulnar diaphysis, in a healthy adult. While existing literature focuses heavily on axial or lower-limb presentations, this report expands the known clinical spectrum, provides evidence of emerging fluoroquinolone resistance, and validates a culture-based management strategy.

## Conclusions

*Salmonella *Typhi osteomyelitis should be actively considered in the differential diagnosis of chronic long-bone infections, even in immunocompetent adults residing in endemic regions. A key clinical takeaway from this case is that standard acute-phase reactants and systemic inflammatory markers are notoriously unreliable in well-sequestered, chronic musculoskeletal conditions, and their normalcy cannot be used to exclude active infection. Definitive diagnosis and successful eradication ultimately require aggressive surgical debridement paired with meticulous, culture-directed antimicrobial therapy.

However, the wider generalizability of our findings is naturally constrained by specific diagnostic and microbiological limitations. The baseline clinical description of this case originally lacked granular parameters regarding exact intraoperative collection depths, anaerobic transport processing speeds, and strict controls over pre-biopsy surgical prophylaxis. Without standardization of these precise specimen logistics, clinicians trying to replicate these outcomes face a higher risk of false-negative cultures or skin commensal contamination, which can mask the true presence of fastidious or atypical pathogens. Furthermore, because* Salmonella* resistance profiles are changing rapidly across South Asia, the successful outcome achieved here using a tier-based cascade reporting protocol highlights that treatment models cannot be generalized as a one-size-fits-all antibiotic regimen. Instead, they must be tailored locally through close collaboration between orthopedic surgeons and clinical microbiology specialists. This case ultimately expands the known anatomical spectrum of extraintestinal salmonellosis and emphasizes that maximizing the global utility of future case reports requires rigorous, standardized reporting of both surgical and microbiological data.
